# Diverse Pharmacological Activities and Potential Medicinal Benefits of Geniposide

**DOI:** 10.1155/2019/4925682

**Published:** 2019-04-16

**Authors:** Yan-Xi Zhou, Ruo-Qi Zhang, Khalid Rahman, Zhi-Xing Cao, Hong Zhang, Cheng Peng

**Affiliations:** ^1^Pharmacy College, Chengdu University of Traditional Chinese Medicine, Chengdu 611137, China; ^2^Library, Chengdu University of Traditional Chinese Medicine, Chengdu 611137, China; ^3^School of Pharmacy and Biomolecular Sciences, Faculty of Science, Liverpool John Moores University, Liverpool L3 3AF, UK; ^4^Institute of Interdisciplinary Medical Sciences, Shanghai University of Traditional Chinese Medicine, Shanghai 201203, China

## Abstract

Geniposide is a well-known iridoid glycoside compound and is an essential component of a wide variety of traditional phytomedicines, for example,* Gardenia jasminoides* Elli (Zhizi in Chinese),* Eucommia ulmoides* Oliv. (Duzhong in Chinese),* Rehmannia glutinosa* Libosch. (Dihuang in Chinese), and* Achyranthes bidentata* Bl. (Niuxi in Chinese). It is also the main bioactive component of Gardeniae Fructus, the dried ripe fruit of* Gardenia jasminoides* Ellis. Increasing pharmacological evidence supports multiple medicinal properties of geniposide including neuroprotective, antidiabetic, hepatoprotective, anti-inflammatory, analgesic, antidepressant-like, cardioprotective, antioxidant, immune-regulatory, antithrombotic, and antitumoral effects. It has been proposed that geniposide may be a drug or lead compound for the prophylaxis and treatment of several diseases, such as Alzheimer's disease, Parkinson's disease, diabetes and diabetic complications, ischemia and reperfusion injury, and hepatic disorders. The aim of the present review is to give a comprehensive summary and analysis of the pharmacological properties of geniposide, supporting its use as a medicinal agent.

## 1. Introduction

**Figure 1:**
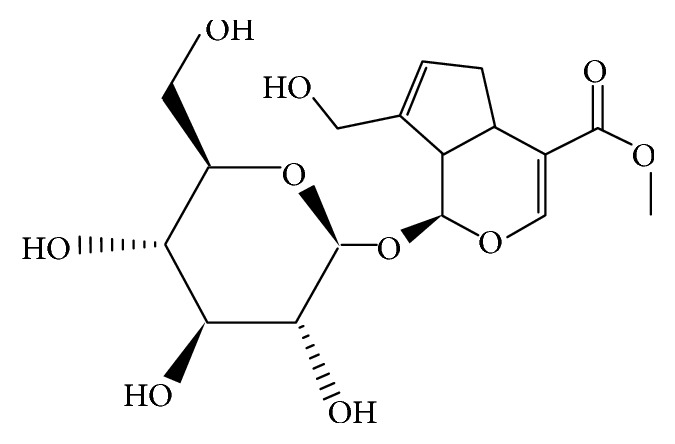
Chemical structure of geniposide.

Geniposide (C_17_H_24_O_10_; Figure 1), a well-known iridoid glycoside compound, is one of the main bioactive components of traditional Chinese medicine Gardeniae Fructus, the dried ripe fruit of* Gardenia jasminoides* Ellis (Zhizi in Chinese). It has been isolated and identified in nearly 40 species of plants and most of which are medicinal herbs [1]. Over the past few decades, there has been a rapid growth in the information available on the pharmacological activities of geniposide. Studies have shown that geniposide displays a wide spectrum of* in vitro* and* in vivo* pharmacological effects, including neuroprotective, antidiabetic, hepatoprotective, anti-inflammatory, analgesic, antidepressant-like, cardioprotective, antioxidant, immune-regulatory, antithrombotic, and antitumoral effects (summarized in Table 1), and these pharmacological effects lay the foundation for geniposide of being a potential therapeutic agent for the treatment of several diseases, such as Alzheimer's disease (AD), Parkinson's disease (PD), diabetes and diabetic complications, ischemia and reperfusion injury, and hepatic disorders. The aim of the present review is to give a comprehensive summary and analysis of the pharmacological properties of geniposide, supporting the potential uses of geniposide as a medicinal agent.

## 2. Pharmacology Actions

### 2.1. Anti-Alzheimer's Disease Activity

AD is the most common neurodegenerative disorder of progressive cognitive decline in the aged population [2]. A number of studies have demonstrated the anti-AD activity of geniposide both* in vitro *and* in vivo*. Formaldehyde is neurotoxic, and long-term overdose increases the risk of AD. An increase in the concentration of endogenous formaldehyde in the brain has been found to correlate with dementia in aging people. In human neuroblastoma SH-SY5Y cells, treatment with geniposide at a concentration of 100 *μ*M ameliorated the morphology of formaldehyde- (0.12 mM) injured cells. Furthermore, geniposide increased the production of the antiapoptotic gene Bcl-2 at both mRNA and protein levels, while decreasing the expression of the proapoptotic gene P53, apoptotic executer caspase-3, and apoptotic initiator caspase-9. Moreover, geniposide elevated the activity of intracellular antioxidants, including superoxide dismutase (SOD) and glutathione peroxidase (GSH-Px) [3]. Incubation of murine N2a neuroblastoma cells with formaldehyde (0.09 mM) for 24 h notably changed cell morphology, decreased cell viability, and promoted apoptosis, and these effects were reversed by supplementation with geniposide (200 *μ*M). In particular, geniposide remarkably ameliorated morphology and cell numbers, increased cell viability, upregulated the expression of Akt and Bcl-2, and lowered the expression of FoxO3 and p53 [4].

Amyloid plaques composed of insoluble amyloid-*β* (A*β*) deposits are a characteristic pathological feature of AD and A*β* aggregated with oligomer is known to be able to induce cellular toxicity as well as cognitive impairment. Incubation with geniposide at 83.8 *μ*M reduced the cytotoxicity of A*β* (10 *μ*M) in rat hippocampal neurons and promoted the outgrowth of neurites both in their length and in the dendrite number per neuron. Geniposide at 5, 100, and 200 *μ*M increased the cell viability of SY5Y-APP695sw cells treated with A*β*_42_ aggregates. Furthermore, geniposide at 100 *μ*M promoted fibrillogenesis and increased the formation of high molecular masses of A*β*_42_ polymers, resulting in a decrease of the quantity and cytotoxicity of A*β*_42_ oligomers [5]. Moreover, geniposide averted A*β*_1−42_-induced cell injury in primary cultured cortical neurons [6]. The receptor for advanced glycation end products- (RAGE-) mediated signaling pathway is related to A*β*-induced pathogenic responses. Geniposide at 50, 100, and 200 *μ*M suppressed RAGE-mediated signaling (activation of extracellular regulated protein kinase (ERK) 1/2 and inhibited kappa B (I*κ*B)/nuclear factor-*κ*B (NF-*κ*B)) and attenuated the production of tumor necrosis factor-*α* (TNF-*α*) and interleukin-1*β* (IL-1*β*) in cultured BV2 microglia cells treated with A*β*_1-42_ (5 *μ*M) [7]. In primary hippocampal neurons, it increased synaptic plasticity through alleviating the A*β*-stimulated downregulation of long-term potentiation and elevating the miniature excitatory postsynaptic current amplitude and frequency. In APP/PS1 transgenic mice, intragastric administration of geniposide at 25 mg/kg for 3 months significantly inhibited RAGE-mediated signaling, lowered the production of TNF-*α* and IL-1*β*, and suppressed cerebral A*β* accumulation. Moreover, geniposide ameliorated learning and memory deficits [8].

A large body of evidence indicates that insulin-degrading enzyme (IDE) plays an essential role in the degradation of A*β*. Geniposide enhanced the expression of IDE in primary cortical neurons, and this effect was reversed by LY294002 (an inhibitor for phosphatidyl inositol 3-kinase, PI3K), GW9662 (an antagonist for peroxisome proliferator-activated receptor *γ*, PPAR*γ*), AG1478 (an antagonist for epidermal growth factor receptor), H89 (an inhibitor for protein kinase A, PKA), and PP1 (an inhibitor for c-Src). Furthermore, it upregulated the phosphorylation of PPAR*γ* and promoted the release of phosphorylated FoxO1 from nuclear fraction to the cytosol. In addition, geniposide directly activated the activity of IDE promoter in PC12 cells [9]. In neuroblastoma SH-SY5Y cells, incubation with geniposide at 1 and 10 *μ*M for 24 h notably augmented the level of IDE protein in a concentration-dependent manner [10].

Furthermore, an increasing number of studies have demonstrated that leptin has protective effect against AD. Geniposide treatment inhibited the level of A*β*_1-42_ both in rat primary cortical neurons (10 *μ*M) and in APP/PS1 transgenic mice (10, 20, and 40 mg/kg, i.g.). Moreover, geniposide at 10 *μ*M induced the phosphorylation of Janus kinase 2 and signal transducer and activator of transcription 3 (STAT3), upregulated the protein level of ADAM10, and downregulated the expression of BACE1 in rat primary cortical neurons, and these effects were reversed by the preincubation with leptin antagonist (50 ng/mL) [11]. Formation of intracellular neurofibrillary tangle from hyperphosphorylated tau is one of the typical hallmark lesions of AD as well. Administration of geniposide enhanced the expression of leptin receptor and diminished the phosphorylation of tau both in rat primary cultured cortical neurons* in vitro* and in APP/PS1 transgenic mice* in vivo.* Additionally, geniposide at 10 *μ*M induced the phosphorylation of Akt at Ser-473 site and glycogen synthase kinase-3*β* (GSK-3*β*) at Ser-9 site in primary cultured cortical neurons, and this effect of geniposide on Akt and GSK-3*β* as well as the effect of geniposide on tau phosphorylation could be inhibited by leptin antagonist (50 nM) [12]. Treatment with geniposide for 4 weeks also attenuated insulin deficiency-induced phosphorylated level of tau and acceleration of GSK-3*β* phosphorylation in the brain of APP/PS1 transgenic mice. It also augmented the effect of insulin on the phosphorylation of tau, Akt, and GSK-3*β* in primary cultured cortical neurons, and these effects were reversed by LY294002 [13]. In streptozotocin- (STZ-) induced AD model rats, injection of geniposide at 50 *μ*M to the lateral ventricle reduced STZ-induced spatial learning deficit, tau phosphorylation, and GSK-3*β* hyperactivity. Additionally, it inhibited STZ-stimulated neural pathology, including accumulation of vesicles in synaptic terminal, paired helical filament-like structures, abnormalities of endoplasmic reticulum (ER), and early stage of apoptosis [14].

Moreover, oxidative stress and mitochondrial dysfunction appear early and contribute to the disease progression in AD. Preincubation with geniposide at 2.5, 5, and 10 *μ*M exerted protective effects against A*β*-induced neuronal injury by blocking mitochondrial dysfunction and oxidative stress. In particular, geniposide ameliorated adenosine triphosphate (ATP) generation, mitochondrial membrane potential levels, cytochrome c oxidase and caspase-3/9 activity, attenuated reactive oxygen species (ROS) production and cytochrome c leakage and suppressed apoptosis in primary cultured mouse cortical neurons incubation with oligomeric A*β*_1-42_ (5 *μ*M) [15]. Intragastric administration of geniposide notably reduced mitochondrial dysfunction in APP/PS1 mice by inhibiting the mitochondrial oxidative stress and elevating the mitochondrial membrane potential and activity of cytochrome c oxidase [16]. Treatment with geniposide at 50 *μ*M for 2 h stimulated the expression of Bcl-2 in PC12 cells induced by H_2_O_2_ (100 *μ*M), and this effect was retarded by preincubation with LY294002. Moreover, geniposide increased the phosphorylation of Akt308, Akt473, GSK-3*β*, and phosphoinositide-dependent protein kinase 1, suggesting that it protects against the oxidative damage induced by H_2_O_2_ in PC12 cells via PI3K signaling pathway [17]. Collectively, geniposide has the potential to be a drug or lead compound for the treatment of AD.

### 2.2. Antidiabetic Activity and Inhibition of Diabetic Complications

Glucose-stimulated insulin secretion (GSIS) from pancreatic *β* cells is one of the key mechanisms in regulating cellular adaptation demands to nutritional and metabolic variations. In rat pancreatic INS-1 cells* in vitro*, geniposide at 5 mM apparently enhanced GSIS, glucose uptake, and ATP production, while at 25 mM it notably decreased these three outcomes. Geniposide time-dependently induced phosphorylation of acetyl-CoA carboxylase, a marker of AMP-activated protein kinase (AMPK) activity, and this effect was notably inhibited by compound C, a selective AMPK inhibitor. Additionally, the knockdown of AMPK protein with AMPK small interfering RNA (siRNA) treatment attenuated geniposide-regulated GSIS, glucose uptake, and ATP production, suggesting that AMPK plays an essential role in the regulatory effect of geniposide on GSIS [18]. In the presence of low (5.5 mM) or moderately high (11 mM) levels of glucose, geniposide at 10 *μ*M enhanced GSIS, glucose uptake, and intracellular ATP level. However, it also exerted a contrary role in the presence of a high (33 *μ*M) level of glucose. Furthermore, geniposide ameliorated the impairment of GSIS stimulated by glucose (33 *μ*M) [19].

Regulation of proteasome-dependent thioredoxin-interacting protein degradation may be a novel strategy to prevent the glucotoxicity in pancreatic *β*-cells. Geniposide accelerated the degradation of thioredoxin-interacting protein via proteasome pathway in high-glucose- (25 mM) treated INS-1 cells. Moreover, geniposide obviously inhibited MG132- (a proteasomal inhibitor) induced enhancement of GSIS, glucose uptake, and metabolism [20]. In another study, it induced insulin secretion, phosphorylation of Akt473 and phosphoinositide-dependent protein kinase 1, and the expression of glucose transporter 2. Furthermore, it decreased the phosphorylation of downstream target GSK-3*β*, and this effect was reversed by LY294002 (10*μ*M) [21]. The glucagon-like-1 receptor (GLP-1R) antagonist exendin (9-39) (200 nM) or knockdown of GLP-1R gene with short hairpin RNA interference offsets the effect of geniposide on insulin secretion stimulated by glucose (5.5 mM) [22]. Moreover, geniposide modulated pyruvate carboxylase expression and the production of intermediates of glucose metabolism [19]. In rat pancreatic islets, geniposide facilitated insulin secretion by activating GLP-1R and the adenylyl cyclase/cAMP signaling pathway, and this effect was blocked via inhibition of PKA. Moreover, geniposide suppressed voltage-dependent potassium channels, and this effect was alleviated via inhibition of GLP-1R or PKA. Furthermore, it prolonged action potential duration and activated Ca^2+^ channels [23].

*β*-cell apoptosis is considered to be a major cause of loss of *β* cells in diabetes. Geniposide significantly inhibited high-glucose- (25 mM) induced cell damage in INS-1 cell. Consistently, it upregulated the protein levels of cell apoptosis-associated enzymes, including heme oxygenase-1 (HO-1) and Bcl-2, while decreasing the protein level of Bax, and these effects were reversed by compound C, but were potentiated by AICAR, an AMPK activator. Furthermore, high glucose-induced cleavage of caspase-3 was prevented by geniposide, and this effect was inhibited by compound C. Additionally, the effects of geniposide on the apoptosis-associated proteins and cell viability were attenuated by AMPK siRNA [24]. Incubation with geniposide at 20 *μ*M for 3 days promoted *β*-cell survival through accelerating *β*-cell proliferation and inhibiting *β*-cell apoptosis in cultured mouse islets after treatment with diabetic stimuli. Geniposide protected *β*-cell by activating Wnt signaling and Akt, promoting expressions of T-cell factor 7-like 2 (TCF7L2) and GLP-1R, suppressing GSK-3*β* activity, and increasing *β*-catenin nuclear translocation, and this effect was obviously reversed by siRNAs against *β*-catenin or by ICG001 (*β*-catenin/TCF-mediated transcription inhibitor). Intragastric administration of geniposide at 100 mg/kg promoted *β*-cell regeneration to normalize blood glucose in high-fat diet and db/db mice. In exocrine cells isolated from mouse pancreas, it induced duct cell differentiation by increasing TCF7L2 expression and activating Janus kinase 2/STAT3 pathway [25]. In INS-1 cells, pretreatment with geniposide for 7 h decreased palmitate-induced *β*-cell apoptosis and active caspase-3 expression, and this effect was reversed by exendin (9-39). Additionally, geniposide ameliorated palmitate-stimulated impairment of GLP-1R signaling by increasing the phosphorylation of Akt and FoxO1 and elevating the expression of pancreatic and duodenal homeobox 1 [26].

Islet amyloid deposition is increasingly seen as a pathogenic feature of type 2 diabetes mellitus, with the deposits containing the unique amyloidogenic peptide islet amyloid polypeptide (IAPP, also known as amylin). Preincubation with geniposide prevented human IAPP- (hIAPP-) induced cell damage in INS-1E cells, and this effect was significantly inhibited by bacitracin, an inhibitor of IDE activity. In addition, it induced the expression of IDE, a key degrading protein of hIAPP, but had no obvious effect on the aggregation of hIAPP, suggesting that geniposide prevents hIAPP-induced cytotoxicity in INS-1E cells via upregulation of IDE expression [27]. In STZ-induced diabetic rats, geniposide at 12.5 and 25 mg/kg reduced the level of A*β*_1-42_ in the hippocampus and at 12.5 mg/kg, it apparently inhibited STZ-induced reduction of the expression of IDE in the hippocampus [10]. Intragastric administration of geniposide at 5, 10, and 20 mg/kg for 4 weeks significantly reduced the levels of cerebral A*β* peptides (A*β*_1-40_ and A*β*_1-42_) in APP/PS1 transgenic mice treated with STZ. Moreover, geniposide lowered the protein levels of ADAM10 (associated with *α*-secretase) and increased the expression of *β*-site APP-cleaving enzyme (BACE1, associated with *β*-secretase) and IDE both in STZ-treated AD mice and in primary cultured cortical neurons. Geniposide at 10 *μ*M augmented the effect of insulin via decreasing A*β*_1-42_ level in primary cultured cortical neurons [28].

Furthermore, intragastric administration of geniposide at 25 mg/kg for 46 days significantly lowered the level of blood glucose, total cholesterol, and triglyceride, while elevating blood insulin levels in STZ-induced diabetic rats [10]. Treatment with geniposide at 200 and 400 mg/kg for 2 weeks reduced the blood glucose, insulin, and triglyceride levels in type 2 diabetic mice induced by a high-fat diet and STZ injection in a dose-dependent manner. Moreover, the expression of hepatic glycogen phosphorylase and glucose-6-phosphatase at mRNA and immunoreactive protein levels, as well as enzyme activity, was decreased in these mice [29]. Geniposide dose-dependently suppressed hepatic glucose production in HepG2 cells and different concentrations of this bioactive compound stimulated AMPK, acetyl coenzyme A synthetase and FoxO1 phosphorylation, and inhibited the activities of phosphoenolpyruvate carboxykinase and glucose-6-phosphatase. These effects were partially counteracted by suppression of AMPK activity via compound C and by inhibition of AMPK*α* expression via siRNA, suggesting that geniposide potentially ameliorates hyperglycemia involving inhibition of hepatic gluconeogenesis via modulation of the AMPK-FoxO1 signaling pathway [30].

In spontaneously obese type 2 diabetic mice, geniposide suppressed body weight and visceral fat accumulation, alleviated abnormal lipid metabolism and intrahepatic lipid accumulation, and ameliorated abnormal glucose tolerance and hyperinsulinemia, suggesting that it has an insulin resistance-alleviating effect [31]. Furthermore, it significantly ameliorated renal function by decreasing levels of serum creatinine, blood urea nitrogen, urinary albumin and elevator renal index and attenuating glomerular basement membrane in STZ-induced diabetic rats. Furthermore, geniposide inhibited monocytes and T-cell infiltration and reduced production of intercellular cell adhesion molecule-1, TNF-*α*, IL-1, and IL-6. Additionally, it suppressed the activation of NF-*κ*B by decreasing expression of NF-*κ*B p65, IKK*α*, and p-I*κ*B-*α* in renal tissue, indicating that it is able to ameliorate structural and functional abnormalities of kidney in diabetic rats [32]. Geniposide at 10-20 *μ*M suppressed adhesion of monocytes to human umbilical vein endothelial cells (HUVECs) stimulated by high glucose (33 mM) in a concentration-dependent manner. At a concentration of 5-40 *μ*M, it lowered high-glucose-stimulated gene and protein expression of vascular cell adhesion molecule 1 and E-selectin. At a concentration of 5-20 *μ*M, it inhibited ROS production and blocked I*κ*B degradation in the cytoplasm and NF-*κ*B translocation from the cytoplasm to the nucleus in HUVECs, suggesting that geniposide may have a role in the treatment for diabetic vascular injury [33].

### 2.3. Anti-Parkinson's Disease Activity

PD is a noncurable chronic neurodegenerative disease at present and geniposide has shown neuroprotective effects in PD models in several* in vivo* and* in vitro* studies. In four 1-methyl-4-phenyl-1,2,3,6-tetrahydropyridine- (MPTP-) (30 mg/kg, i.p.) induced acute PD model mice, intraperitoneal injection of geniposide at 100 mg/kg for 8 days ameliorated bradykinesia and movement balance and improved locomotor and exploratory activity. In addition, MPTP treatment reduced tyrosine hydroxylase positive neuron, lessened the level of Bcl-2, and increased the level of Bax, the ratio of Bax/Bcl-2, and the activation of caspase-3 in the substantia nigra. Nevertheless, these alterations were reversed by geniposide, suggesting that the neuroprotective activity of geniposide is associated with upregulation of growth factor signaling and reduction of apoptosis [34]. In human neuroblastoma SH-SY5Y cells treated with N-methyl-4-phenylpyridinium iodide* in vitro* and PD model mice induced by MPTP (20mg/kg)* in vivo*, geniposide administration decreased microRNA-21 (miR-21) level, increased lysosome-associated membrane protein 2A (LAMP-2A) level, and lessened *α*-synuclein protein expression. LAMP-2A mediated the regulation of *α*-synuclein by miR-21. The high expression of LAMP-2A retarded the upregulation of *α*-synuclein induced by miR-21 mimic. The downregulating effect of geniposide on *α*-synuclein was reversed by miR-21 mimics/agomir, while it was augmented by miR-21 inhibitor, suggesting that geniposide exerts its neuroprotective effects through reducing *α*-synuclein expression via the miR-21/LAMP-2A axis in PD models [35].

### 2.4. Inhibition of Ischemia and Reperfusion Injury

The protective effect of geniposide against ischemic brain injury has been investigated in* in vitro *and* in vivo* models and increasing number of studies have focused on its therapeutic role in ischemic cerebral disease. Oral administration of geniposide at 50 mg/kg for 30 days exhibited an improved effect on learning and memory ability and neural protective effects in chronic cerebral ischemia model rats induced by permanent occlusion of bilateral common carotid arteries [36]. Geniposide at 75 mg/kg regulated antiapoptotic functions and suppressed blood-brain barrier leakage/haemorrhage by increasing GluN2A-containing* N*-methyl-D-aspartate receptor expression in transient middle cerebral artery occlusion rats, and these effects could be counteracted by GluN2A antagonist NVP-AAM077, but not the GluN2B inhibitor ifenprodil. Furthermore, the protective effect of geniposide was attributed to the upregulation of GluN2A-dependent survival signals, including p-Akt, p-ERK, and postsynaptic density protein-95, suggesting that it protects neurons against postischaemic neurovascular injury involving activation of GluN2A/Akt/ERK pathways [37]. Preadministration of geniposide at 5, 10, and 20 mg/kg protected Sprague-Dawley rats against hepatic ischemia/reperfusion injury by decreasing alanine transaminase (ALT), aspartate transaminase (AST), lactate dehydrogenase, Bax, IL-6, monocyte chemotactic protein 1, and TNF-*α* levels, increasing Bcl-2, PI3K, p-Akt, and mammalian target of rapamycin (mTOR) expressions, and lowering inflammatory cell infiltration, cellular swelling, and vacuolar degeneration [38].

Furthermore, treatment with geniposide at 15, 30, and 60 mg/kg through tail vein injection lowered the infarct volume and suppressed the activation of microglial cells in ischemic penumbra of ischemia/reperfusion-injured rats induced by middle cerebral artery occlusion. In oxygen-glucose deprivation- (OGD-) treated rat microglial cells, geniposide at 12.5, 25, and 50 *μ*g/mL restrained the increase of cell viability and release of TNF-*α*, IL-1*β*, IL-6, IL-8, and IL-10. Furthermore, it lowered OGD-induced increase of Toll-like receptor 4 (TLR4) mRNA and protein levels. Geniposide at 25 and 50 *μ*g/mL attenuated the phosphorylation of ERK, I*κ*B, and p38 and suppressed nuclear transcriptional activity triggered via NF-*κ*B p65 [39]. Additionally, it significantly suppressed the upregulated mRNA and protein expression of P2Y_14_ and inhibited the phosphorylation of Raf-1, mitogen-activated protein kinase kinase 1/2 (MEK1/2), and ERK1/2 both in OGD-induced rat brain microvascular endothelial cells (BMECs) and in UDP-glucose stimulated BMECs. Moreover, it notably reduced the production of IL-8, IL-1*β*, and monocyte chemotactic protein 1 in BMECs treated with OGD [40]. In rat hippocampal slice culture, it attenuated OGD-induced neuronal cell death. Besides, geniposide exhibited a greater protective effect on the granule cell layer than on the pyramidal cell layer including CA 1 and CA 3 region in the hippocampus [41]. In addition, it increased the viability of neonate rat primary cortical neuron with sodium dithionite-induced OGD/reperfusion [42].

In H9c2 cells, pretreatment with geniposide in gradient concentrations of 2.5, 5, 10, 20, 40, 80, 160, and 320 *μ*M for 30 min elevated cell viability, lowered lactate dehydrogenase levels, and suppressed cardiomyocyte apoptosis induced by hypoxia/reoxygenation. Furthermore, it lessened oxidative stress products (ROS/reactive nitrogen species and malondialdehyde), elevated antioxidative enzyme (total SOD) level, improved mitochondrial morphology, and inhibited mitochondrial calcium overload and depolarization of mitochondrial membrane, thus reversing mitochondrial dysfunction. Moreover, geniposide upregulated Bcl-2 and p-Akt^serine473^ levels, downregulated Bax level, caspase-3 mRNA expression, and the protein expression of cleaved caspase-3 and cytochrome-c, and these effects were partially counteracted by exendin (9-39) or LY294002, suggesting that it inhibits hypoxia/reoxygenation-induced myocardial apoptosis through reversing mitochondrial dysfunction, an effect in part due to activation of GLP-1R and PI3K/Akt signaling pathway [43].

### 2.5. Hepatoprotective Activity

Geniposide modified abnormal metabolism induced by ethanol exposure by alleviating disorders relating to amino acid metabolism and the oxidative stress state in alcohol-induced liver injury model mice [44]. Intragastric administration of geniposide at 20, 40, and 80 mg/kg notably inhibited the acute alcohol-induced increase of both serum ALT/AST and hepatic lipid peroxidation levels. Furthermore, it increased the hepatic GSH, GSH transferase, GSH-Px, CuZn-SOD, and catalase (CAT) levels. Moreover, geniposide increased hepatic mRNA expressions of CuZn-SOD and CAT, indicating that it protects against acute alcohol-induced liver injury by upregulating the expression of the main antioxidant enzymes and thus improved alcohol-induced oxidative stress injury in the liver [45]. Treatment with geniposide at 25, 50, and 100 mg/kg for 6 weeks ameliorated liver histology by decreasing the elevated liver index (liver weight/body weight); serum ALT and AST in high-fat diet caused nonalcoholic steatohepatitis in model rats. Geniposide reduced total cholesterol, triglycerides, and free fatty acids in serum and liver. Additionally, it inhibited cytochromes P450 2E1 expression and elevated PPAR*α* expression [46].

In Sprague-Dawley rats, intragastric administration of geniposide at 25, 50, and 100 mg/kg blocked *α*-naphthylisothiocyanate- (ANIT-) induced changes in serum markers for liver injury. Moreover, geniposide decreased basolateral bile acids uptake by repressing organic anion transporting polypeptide 2. Furthermore, it markedly elevated canalicular bile acids secretion via bile acids export pump, subsequently stimulating bile flow during cholestasis. Meanwhile, geniposide significantly increased mRNA levels of organic solute transporter *β*. In addition, farnesoid X receptor, pregnane X receptor, and small heterodimer partner were activated, indicating the attenuation of ANIT-induced hepatotoxicity and cholestasis in rat via regulation of enzymes and transporters responsible for bile acids homeostasis [47]. In ICR mice, treatment with geniposide at 50 mg/kg for 5 days mitigated ANIT-induced cholestasis and liver injury. Furthermore, it normalized the altered gene transcription related to bile acid metabolism and transport and suppressed the activation and expression of STAT3 [48].

Pretreatment of mice with geniposide at 20, 40, and 80 mg/kg for 7 days by intragastrical administration attenuated* Tripterygium* glycosides- (270 mg/kg) induced elevation of serum ALT/AST, hepatic malondialdehyde, and proinflammatory cytokine TNF-*α* level. Moreover, it increased* Tripterygium* glycosides-induced downregulation of biomarkers, including hepatic GSH level; activities of GSH transferase, GSH-Px, SOD and CAT, and anti-inflammatory cytokine IL-10 were observed. In addition, it promoted the mRNA expression of hepatic transforming growth factor-beta1 (TGF-*β*1) [49]. In human HepG2 hepatocytes, geniposide suppressed palmitate-induced ER stress and inhibited hepatic lipid accumulation via secretion of apolipoprotein B and associated triglycerides and cholesterol. Moreover, lysosomal enzyme activities including V-ATPase were significantly increased, and these effects were reversed by bafilomycin, a V-ATPase inhibitor, suggesting that geniposide enhances lysosomal activity, resulting in reduction of ER stress and hepatic dyslipidemia [50]. Besides, it inhibited palmitate-induced cell death via enhancement of lysosome activity [51]. In AML12 cells, geniposide apparently blocked TGF-*β*1-induced mRNA and protein expression of type-I collagen and phosphorylation of Smad2/3; ERK and Akt were significantly repressed [52]. Taken together, geniposide might be a potential therapeutic candidate for treatment of liver diseases.

### 2.6. Anti-Inflammatory Activity

Geniposide is reported to relieve the secondary hind paw swelling and arthritis index and reduce phosphorylated c-Jun N-terminal kinase (JNK) expression and T helper 17 cell cytokines, while increasing regulatory T-cell cytokines in mesenteric lymph node lymphocytes (MLNL) and peripheral blood lymphocytes of adjuvant arthritis rats [53]. Oral administration of geniposide at 30, 60, and 120 mg/kg attenuated histopathologic changes, decreased IL-2 level, and increased the level of IL-4 and TGF-*β*1 in MLNL. Moreover, it elevated the expression of *β*2-adrenergic receptor and lowered the expression of G protein-coupled receptor kinase 2, *β*-arrestin-1 and *β*-arrestin-2, and level of cyclic adenosine monophosphate of MLNL [54]. Furthermore, it facilitated the recovery of arthritis and suppressed the colonic inflammation damage through reducing the expression level of TNF-*α*, IL-1, and IL-6, elevating the production of IL-10 and blocking the expression of phospho-p38 related proteins in fibroblast-like synoviocyte (FLS) [55]. In collagen-induced arthritis rat, oral administration of geniposide at 33, 66, and 132 mg/kg attenuated histopathologic changes of MLN and decreased the level of IL-6 and IL-17, while the levels of IL-4 and TGF-*β*1 in MLNL were increased. Additionally, geniposide promoted the proliferation capability of MLNL and inhibited the expressions of p-Raf, p-MEK, and p-ERK1/2 in MLNL [56], and these results might highlight the anti-inflammatory effect and possible mechanisms of geniposide in MLNL and FSL of rheumatoid arthritis.

In LPS-induced mastitis model mice, intraperitoneal administration of geniposide at 2.5, 5, and 10 mg/kg notably attenuated the infiltration of inflammatory cells. Besides, it decreased the expression of TNF-*α*, IL-1*β*, and IL-6 both in LPS-induced mastitis model mice (2.5, 5, and 10 mg/kg) and in LPS-stimulated primary mouse mammary epithelial cells (25, 50, and 100 *μ*g/mL). In addition, it inhibited the phosphorylation of I*κ*B-*α*, NF-*κ*B, p38, ERK, and JNK and the expression of TLR4, indicating that geniposide inhibits inflammation through regulating TLR4 expression, resulting in affection of the downstream NF-*κ*B and mitogen-activated protein kinase (MAPK) signaling pathways [57]. In C57BL/6 mice after* Helicobacter pylori* infection, geniposide inhibited vacuolating cytotoxin A gene expression, downregulated the serum levels of IFN-*γ*, IL-1*β*, and immunoglobulins A and M and decreased the inflammatory maker cyclooxygenase 2 (COX-2) [58]. Furthermore, it notably inhibited proinflammatory cytokines through regulating NF-*κ*B and PPAR*γ* pathways in dextran sulfate sodium-induced experimental colitis in mice and LPS-triggered inflammation in Caco-2 cells. Besides, geniposide notably regulated the expressions of zonula occludens-1 and occludin attenuated body weight loss, disease activity index, colon length shortening, and colonic pathological damage in dextran sulfate sodium-induced colitis model mice [59].

In another study, administration of geniposide at 25 and 50 mg/kg notably elevated the decreased body weight and improved experimental colitis and related symptoms in 2,4,6-trinitrobenzene sulfonic acid-induced experimental ulcerative colitis model rats. Geniposide inhibited inflammatory cytokine release, including TNF-*α*, IL-1*β*, and IL-6, and neutrophil infiltration (myeloperoxidase activity) in the colon. At a concentration of 25-100 *μ*g/mL, geniposide improved LPS-induced endothelial barrier dysfunction via the dose-dependent upregulation of transepithelial electrical resistance in Caco-2 cells. Furthermore, it lowered the protein expression of NF-*κ*B, COX-2, inducible nitric oxide synthase (iNOS), and myosin light-chain kinase (MLCK), increased the expression of tight junction proteins (occludin and zonula occludens-1), and accelerated AMPK phosphorylation both* in vivo* and* in vitro*. Geniposide-reduced MLCK protein expression was antagonized by both AMPK siRNA transfection and AMPK overexpression, suggesting that geniposide improves barrier dysfunction via AMPK-mediated inhibition of the MLCK pathway. Taken together, geniposide improved 2,4,6-trinitrobenzene sulfonic acid-induced colitis by attenuating inflammation and regulating the disrupted epithelial barrier function via activating the AMPK signaling pathway [60]. Moreover, it alleviated inflammation in acute liver injury model mice and in LPS-stimulated THP-1 cells. Geniposide decreased the expressions of Methyl-CpG binding protein 2, Sonic hedgehog, and GLIS family zinc finger 1, while elevating Patched1 expression, suggesting that it may be an effective modulator of Methyl-CpG binding protein 2-hedgehog pathway during the pathogenesis of inflammation [61]. Pretreatment with geniposide at 20, 40, and 80 mg/kg reduced inflammatory cells and total protein concentration in the bronchoalveolar lavage fluid of LPS-induced acute lung injury model mice and furthermore reduced levels of inflammatory mediators, including TNF-*α*, IL-6, IL-10 [62], and IL-1*β* were observed [63]. In addition, it suppressed LPS-induced alveolar wall changes, alveolar haemorrhage, and neutrophil infiltration in lung tissue by reducing myeloperoxidase activity [62].

In primary mouse macrophages, geniposide significantly suppressed the expression of TLR4 and the production of TNF-*α*, IL-6, and IL-1*β* induced by LPS and prevented LPS-induced phosphorylation of I*κ*B-*α*, p65, p38, ERK, and JNK. Moreover, it inhibited the LPS-induced IL-8 production in mTLR4 and mMD-2 cotransfected HEK293 cells [63]. In murine microglial N9 cells, pretreatment with geniposide at 40, 80, and 160 *μ*g/mL for 20 h suppressed the activation of cells and restrained the overproduction of NO, intracellular ROS, and the expression of iNOS induced by LPS (1 *μ*g/mL). Geniposide at 80 and 160 *μ*g/mL inhibited LPS-induced drop-off of I*κ*B and phosphorylation of p38 and ERK1/2 [64]. In murine macrophage RAW 264.7 cells, it decreased the expression of TNF-*α*, IL-1, IL-6, and IL-1*β* and furthermore suppressed activation of NF-*κ*B and expression of TLR4 at both mRNA and protein levels [65, 66]. Pretreatment with geniposide at 40, 80, and 160 *μ*g/mL for 1 h dose-dependently exerted anti-inflammatory effects through preventing the LPS-stimulated NF-*κ*B, MAPK, and activator protein-1 signaling pathway activation, subsequently blocking the overexpression of the inducible enzymes iNOS and COX-2. Furthermore, geniposide inhibited the expression and release of inflammatory-associated mediators TNF-*α*, IL-6, prostaglandin E_2_, and NO [67]. In HUVECs, treatment with geniposide at 25-100 *μ*g/mL for 24 h evidently decreased LPS-induced transcription and translation levels of IL-6 and IL-8 and blocked LPS-induced HUVEC migration and U937 monocyte adhesion to HUVECs. Moreover, geniposide inhibited the activation of NF-*κ*B, degradation of I*κ*B-*α*, and phosphorylation of p38 MAPK and ERK1/2 in HUVECs stimulated by LPS, indicating that it attenuates LPS-stimulated IL-6 and IL-8 production in HUVECs through inhibiting p38 MAPK and ERK1/2 signaling pathways [68]. In summary, geniposide has the potential to be a therapeutic agent in the treatment of inflammatory diseases, including mastitis, colitis, and arthritis.

### 2.7. Antidepressant-Like Activity

Treatment with geniposide at 25, 50, and 100 mg/kg counteracted chronic unpredictable mild stress-induced behavioral abnormalities by increasing sucrose intake, improving crossing and rearing behavior in open field test, and by shortening immobility and prolonging swimming time in forced swimming test. Moreover, geniposide ameliorated chronic unpredictable mild stress-induced hyperactivity of hypothalamus-pituitary-adrenal axis through reducing corticosterone serum level, adrenal gland index, and hypothalamic corticotrophin-releasing hormone mRNA expression. Additionally, it increased the expression of hypothalamic glucocorticoid receptor *α* at both mRNA and protein levels in paraventricular nucleus, suggesting that geniposide ameliorates the impaired glucocorticoid receptor *α* negative feedback on corticotrophin-releasing hormone expression and hypothalamus-pituitary-adrenal axis and indicating that the therapeutic effects of geniposide are essentially similar to fluoxetine [69]. In STZ-induced diabetic mice, intragastric administration of geniposide at 50 and 100 mg/kg alleviated the elevated blood sugar and immobility time in forced swimming test. Furthermore, it upregulated brain-derived neurotrophic factor levels and increased the mRNA expression of brain-derived neurotrophic factor and tropomyosin-related kinase B in hippocampus of diabetic mice, indicating that geniposide alleviates depression-like behavior in STZ-induced diabetic mice and suggesting that its mechanisms may partially be associated with upregulating brain-derived neurotrophic factor expression in brain [70].

### 2.8. Antioxidant Activity

In primary cultured hippocampal neurons, pretreatment with geniposide increased the HO-1 expression and subsequently inhibited cell apoptosis stimulated by 3-morpholinosydnonimine hydrochloride (SIN-1, 1mM). Additionally, geniposide (10 *μ*M) enhanced the nuclear translocation of nuclear factor-E2-related factor 2 and activation of PI3K in the presence of SIN-1-induced oxidative stress, and these effects could be retarded by LY294002 (10 *μ*M) and Zinc protoporphyrin (an inhibitor of HO-1, 10 *μ*M), indicating that it protects primary hippocampal neurons from oxidative stress by regulating nuclear factor-E2-related factor 2 translocation and downstream antioxidant protein HO-1 expression via PI3K/Akt signaling pathway [71]. In another study, geniposide-induced luciferase activity enhanced after transfecting PC12 cells with the AB1 enhancer from the HO-1 gene, but not in the PC12 cells whose GLP-1R gene was disrupted. Moreover, the neuroprotective activity of geniposide in PC12 cells was blocked by the prevention of HO-1 activity via Sn-protoporphyrin IX or short hairpin RNA-mediated knockdown of GLP-1R, suggesting that GLP-1R plays a critical role in geniposide-induced HO-1 expression to attenuate oxidative insults in PC12 cells [72].

### 2.9. Antiapoptotic Activity

Geniposide significantly reduced the percentage of sodium nitroprusside-stimulated chondrocytes in G0/G1 phase and elevated percentage in S phase and G2/M phase* in vitro*. In addition, it suppressed the apoptosis of chondrocyte and the concentration of NO in the culture supernatants. Moreover, it impacted sodium nitroprusside-induced apoptosis of chondrocyte by reducing the concentration of NO and promoting proliferation of chondrocytes, and this effect was a probable and important mechanism of geniposide preventing osteoarthritis [73]. Intraperitoneal administration of geniposide at 2.5, 5, and 10 mg/kg inhibited mammary gland apoptosis, Bax expression, and the phosphorylation of caspase-3 cleavage and p53 and augmented Bcl-2 expression in LPS- (0.2 mg/mL) induced mastitis model mice. In LPS- (1 *μ*g/mL) induced primary mouse mammary epithelial cells, geniposide at 25, 50, and 100 *μ*g/mL downregulated the ratio of dead cells in a concentration-dependent manner. Consistently, geniposide attenuated the expressions of Bax and TLR4, caspase-3 cleavage, and the phosphorylation of p53, while increasing Bcl-2 expression, suggesting that the antiapoptotic property of geniposide is due to its modulation of TLR4 and apoptosis-related factors (p53, Bax, Bcl-2, and caspase-3) in LPS-induced mouse mastitis [74]. Additionally, it significantly ameliorated cell viability and morphology, reduced the expression of Bax, P53, and caspase-9, and upregulated the expression of Bcl-2 in CoCl_2_-stimulated PC12 cells [75].

### 2.10. Immune-Regulatory Activity

Oral administration of geniposide at 100 mg/kg for 12 weeks showed immune-regulatory effects and inhibited the formation of atherosclerotic lesions via lowering the dendritic cell numbers and prevented dendritic cell maturation in bone marrow and infiltration into lesions in ApoE-knockout mice [76]. Furthermore, it increased FoxP3 expression, elevated the number and function of regulatory T cells, and improved the atherosclerotic lesions progression partly via lipid regulation and immunoregulation in ApoE-/- mice fed a high-cholesterol diet [77].

### 2.11. Antisepsis Activity

In RAW264.7 cells, geniposide could directly bind and neutralize LPS and it dose-dependently blocked LPS-induced cytokines release, reduced the upregulated TNF-*α* and TLR4 mRNA expression, and inhibited the phosphorylation of p38 MAPK.* In vivo*, treatment with geniposide at 40 mg/kg protected mice challenged with lethal heat-killed* Escherichia coli* and downregulated the level of serum endotoxin in endotoxemia mice [78]. Geniposide exhibited high binding affinity for lipid A, the bioactive center of lipopolysaccharide (LPS). In LPS-induced sepsis model mice, geniposide lowered LPS levels and release of IL-6 and TNF-*α* induced by LPS and exhibited protective activities on liver, heart, and lung [79].

### 2.12. Other Biological Activities

#### 2.12.1. Cardioprotective Activity

Intragastric administration of geniposide at 50 mg/kg for 7 weeks inhibited cardiac hypertrophy induced by constriction of the transverse aorta or by isoprenaline in mice. It also activated AMPK*α* and decreased phosphorylated level of mTOR, ERK, and protein kinase double-stranded RNA-dependent-like ERK in hypertrophic heart induced by constriction of the transverse aorta. Furthermore, geniposide attenuated the expressions of glucose regulated protein 78, X-box binding protein 1, and activating transcription factor 6. GLP-1R blockade retarded the antihypertrophic response and activation of AMPK*α* by geniposide. Besides, knockdown of GLP-1R impeded the inhibitory effects of geniposide on cardiac hypertrophy* in vivo*, suggesting that it protects against cardiac hypertrophy via GLP-1R and AMPK*α* and indicating that it may be a potential therapeutic candidate for cardiac hypertrophy [80].

#### 2.12.2. Antinociceptive Activity

Geniposide concentration-dependently exerted the protective effect against hydrogen peroxide-caused oxidative damage in PC12 and HEK293 cells expressing rat and human GLP-1R, but not in HEK293T cells that do not express GLP-1R. Oral and subcutaneous administration of geniposide dose-dependently inhibited formalin-induced tonic response, but not the acute flinching response. Intrathecal administration of geniposide induced antinociception in a dose-dependent manner, and this effect was completely reversed by spinal exendin (9-39), siRNA/GLP-1R, and cyclic AMP/PKA pathway inhibitors, suggesting that geniposide exerts antinociception during persistent pain by activating the spinal GLP-1R [81].

#### 2.12.3. Apoptotic Activity

*In vitro*, geniposide suppressed adjuvant-induced arthritis (AIA) FLS proliferation in a dose-dependent manner and induced typical apoptotic morphological characteristics including nuclear shrinkage and chromatin condensation and notably promoted the apoptosis rate of AIA FLS. Furthermore, it reduced Bcl-2 mRNA level and elevated Bax and caspase-3 mRNA levels and lowered protein level of phosphorylated-ERK1/2, indicating that geniposide induced AIA FLS apoptosis by regulating the apoptosis-related gene expressions and preventing ERK signal pathway [82].

#### 2.12.4. Antithrombotic Activity

Geniposide at 20 mg/kg suppressed thrombin/collagen-induced platelet aggregation with 52.8%/26.2% aggregation rate in rats* ex vivo*. At a concentration of 10-40 mg/kg, it inhibited venous thrombosis induced by tight ligation of the inferior vena cava in mice, and the *ED*_50_ value was 18.4 mg/kg [83].

#### 2.12.5. Antiviral Activity

Geniposide inhibited enterovirus 71 replication and viral internal ribosome entry site activity, suggesting that it may be a potential chemopreventive agent against enterovirus 71 infections [84].

#### 2.12.6. Promelanogenesis Activity

In norepinephrine-treated normal human epidermal melanocyte, geniposide enhanced melanogenesis via stem cell factor/c-kit signalling in which the expression of c-kit receptor was increased [85].

#### 2.12.7. Antitumoral Activity

Geniposide at 50 and 100 *μ*M was able to stabilize covalent attachments of the topoisomerase I subunits to DNA at sites of DNA strand breaks, generating cleavage complexes intermediates so being active as poisons of topoisomerase I, suggesting that DNA damage induced by topoisomerase I poisoning may be one of the possible mechanisms of antitumoral activity displayed by geniposide [86].

## 3. Conclusion

Geniposide, the major iridoid glycoside of Gardeniae Fructus, has been isolated from nearly 40 species of plants and most of which are used as traditional phytomedicines. Increasing body of pharmacological evidence has emerged for the multiple bioactive functions of geniposide, including neuroprotective, antidiabetic, hepatoprotective, anti-inflammatory, analgesic, antidepressant-like, cardioprotective, antioxidant, immune-regulatory, antithrombotic, and antitumoral effects. The active mechanisms are related to enhancement of SOD, GSH, CAT, IDE, PPAR*γ*, and FoxO1, inhibition of ROS, NO, ALT, AST, and *α*-synuclein, and regulation of signaling pathways such as AMPK, NF-*κ*B, and PI3K/Akt.* In vivo* and* in vitro* studies showed that geniposide could pass through the blood-brain barrier with poor permeability [87, 88]. However, the poor blood-brain barrier permeability of geniposide could be significantly improved via structural modification [89] or prescription with a messenger agent, such as borneol and muscone [88, 90]. Collectively, it has been suggested that geniposide may be a drug or lead compound for the prophylaxis and treatment of several diseases, such as AD, PD, diabetes and diabetic complications, ischemia and reperfusion injury, and hepatic disorders.

However, the study of geniposide is only in the primary stage since the molecular mechanisms and targets underlying pharmacological effects are not yet clearly defined. At present, its clinical application is mainly in the form of traditional Chinese preparations instead of geniposide monomer. In addition, toxicity of geniposide has also been reported, for example, hepatotoxicity [91, 92]; oral chronic toxicity of geniposide in rats has also been reported which resulted in the serum biochemistry, urinalysis, haematological parameters, and relative organ weights being affected. Furthermore, its usage has caused noticeable pathological abnormalities in liver and kidney tissues [93]. Besides, it shows two-way regulation on certain effects, which may be related to the dosage and experimental subjects, but the specific reasons are not very clear. Thus, further studies are required to verify and develop the safe use of geniposide.

## Figures and Tables

**Table 1:** Summary of the *in vitro* and *in vivo* evidence for the biological activities of geniposide.

Pharmacological effect	*In vitro* evidence	*In vivo* evidence
Anti-Alzheimer's disease activity [2–17]	(i) Neuroprotective effects in neuroblastoma SH-SY5Y cells, N2a neuroblastoma cells, primary hippocampal neurons, BV2 microglia cells, primary cortical neurons, and PC12 cells	(i) Inhibits RAGE-mediated signaling, enhances leptin receptor expression, reduces tau phosphorylation, mitochondrial dysfunction and cerebral A*β* peptide level in mice (ii) Reduced STZ-induced spatial learning deficit, tau phosphorylation, GSK-3*β* hyperactivity, and neural pathology in rats

Antidiabetic activity and inhibition of diabetic complications [10, 18–33]	(i) Regulates GSIS, antagonizes cell injury, and counteracts lipotoxicity in INS-1 cells (ii) Regulates GSIS in primary pancreatic *β*-cells (iii) Promotes *β*-cell survival in cultured mouse islets (iv) Prevents cytotoxicity in INS-1E cells (v) Suppresses hepatic glucose level in HepG2 cells (vi) Inhibits cell adhesion in HUVECs	(i) Promotes *β*-cell regeneration and prevents metabolic disease status in mice (ii) Modulates blood glucose, insulin, total cholesterol and triglyceride, decreases cerebral A*β* peptide level, and reduces development of diabetic nephropathy in rats

Anti-Parkinson's disease activity [34, 35]	(i) Prevents *α*-synuclein expression in SH-SY5Y cells	(i) Enhances growth factor signaling, reduces apoptosis, and inhibits *α*-synuclein expression in mice

Inhibition of ischemia and reperfusion injury [36–43]	(i) Attenuates inflammatory responses in rat microglial cells and brain microvascular endothelial cells (ii) Attenuates neuronal cell death in rat hippocampal slice culture (iii) Increases rat primary cortical neuron viability (iv) Inhibits myocardial apoptosis in H9c2 cells	(i) Improves learning and memory ability, attenuates postischaemic neurovascular injury, and prevents hepatic injury and microglial cells activation in rats

Hepatoprotective activity [44–52]	(i) Enhances lysosomal activity in HepG2 cells (ii) Suppresses epithelial-mesenchymal transition in AML12 cells	(i) Prevents liver damage and cholestasis in mice (ii) Attenuates hepatic steatosis and cholestasis in rats

Anti-inflammatory activity [53–68]	(i) Inhibits inflammation in primary mouse mammary epithelial cells, Caco-2 cells, THP-1 cells, primary mouse macrophages, HEK293 cells, N9 microglial cells, RAW264.7 cells, and HUVECs	(i) Inhibits inflammation in rats and mice

Antidepressant-like activity [69, 70]		(i) Alleviates depression-like behavior in rats and mice

Antioxidant activity [71, 72]	(i) Increases HO-1 expression in primary hippocampal neurons and PC12 cells	

Anti-apoptotic activity [73–75]	(i) Inhibits apoptosis in chondrocytes, primary mouse mammary epithelial cells, and PC12 cells	(i) Inhibits apoptosis in mice

Immune-regulatory activity [76, 77]		(i) Induces regression of atherosclerosis in mice

Antisepsis activity [78, 79]	(i) Binds and neutralizes LPS in RAW264.7 cells	(i) Protects mice challenge with lethal heat-killed *E. coli*(ii) Neutralizes LPS and decreases serum endotoxin level in mice

Cardioprotective activity [80]		(i) Inhibits cardiac hypertrophy in mice

Antinociceptive activity [81]		(i) Produces antinociception during persistent pain in rats

Apoptotic activity [82]		(i) Induces fibroblast-like synoviocytes apoptosis in rats

Antithrombotic activity [83]	(i) Inhibits platelet aggregation	(i) Inhibits venous thrombosis in mice

Antiviral activity [84]	(i) Inhibits enterovirus 71 infections	

Promelanogenesis activity [85]	(i) Enhances melanogenesis in human epidermal melanocyte	

Antitumoral activity [86]	(i) Induces the formation of DNA cleavage complex	
